# Strategies to tackle non-communicable diseases in Afghanistan: A scoping review

**DOI:** 10.3389/fpubh.2023.982416

**Published:** 2023-02-22

**Authors:** Narges Neyazi, Ali Mohammad Mosadeghrad, Mahnaz Afshari, Parvaneh Isfahani, Najibullah Safi

**Affiliations:** ^1^International Campus, School of Public Health, Tehran University of Medical Sciences, Tehran, Iran; ^2^Health System Development, World Health Organization Country Office, Kabul, Afghanistan; ^3^Health Information Management Research Center, School of Public Health, Tehran University of Medical Sciences, Tehran, Iran; ^4^Social Determinants of Health Research Center, Saveh University of Medical Sciences, Saveh, Iran; ^5^School of Public Health, Zabol University of Medical Sciences, Zabol, Iran

**Keywords:** Afghanistan, non-communicable diseases, prevention and control, strategies, hypertension, diabetes, heart diseases, risk factors

## Abstract

Non-communicable diseases (NCDs) and their risk factors are the leading cause of death worldwide and contribute to 74.3% of deaths globally in 2019. The burden of NCDs is escalating in Afghanistan. Currently, every seconds, people in Afghanistan are dying of NCDs. Addressing this challenge in Afghanistan needs effective and practical interventions. This study aimed to identify the strategies developed and implemented in countries with low non-communicable premature death. To conduct a scoping review, we followed the six-step Arksey and O'Malley protocol and searched for eligible articles on eight international databases and the gray literature. The study followed the Preferred Reporting Items for Systematic review and Meta-Analysis (PRISMA) guidelines. The inclusion criteria were English documents and evidence produced up to 30 November 2021 for the control of NCDs. We excluded incomplete texts, duplicates, and dissertations due to lack of access. We used EndNote X9 and MaxQDA software for data management and analysis. We conducted content analysis for this study. A total of 122 documents developed between 1984 and 2021 met the inclusion criteria. We identified 35 strategies from which the most used strategies were related to unhealthy diets and smoking cessation programs. Canada (26.4%), Korea (19.8%), and the United Kingdom (19%) have the most publications on the control and prevention of NCDs among the countries included in the study. Most strategies were implemented over 2 years (41%). This study recommends specific interventions to control and prevent NCDs for the main risk factors of tobacco use, unhealthy diet, physical inactivity, and the main non-communicable diseases such as heart diseases, cancers, diabetes, and chronic obstructive pulmonary diseases. Afghanistan Ministry of Public Health, the WHO country office, and other involved stakeholders can use the findings of this review to design and implement strategies for controlling and preventing NCDs in Afghanistan. International organizations such as the World Health Organization, United Nations Agencies, the World Bank, and other involving communities should invest in strengthening good health governance in Afghanistan. The Afghan Government should focus on promoting and funding health literacy among the public and self-care to control and prevent NCDs.

## 1. Introduction

Non-communicable diseases (NCDs) are the major cause of death worldwide. The four prominent diseases are cardiovascular diseases, cancers, diabetes, and chronic obstructive pulmonary diseases (COPD) (1). NCDs and their common risk factors were the cause of 74.3% of deaths globally in 2019 (2). Importantly, three-quarters of these deaths and 80% of premature deaths occurred in low- and middle-income countries (LMICs) (3). Four risk factors are the common contributors to these diseases: smoking, harmful use of alcohol, physical inactivity, and unhealthy diet (4). As these factors can be influenced and contribute to reducing premature death, it is of utmost importance to focus on measures to reduce them.

Afghanistan is classified as a low-income country in which the health indicators have the worst situation after some African countries such as Sierra Leone, Central African Republic, Chad, Guinea-Bissau, and Nigeria in the world (5). This country was positioned at 169 out of 188 in the low development category (6). The total population of Afghanistan is 32,527,000 and the country's size is 625,864 km^2^. More than half of Afghanistan's population is male (51.3%) (7) and the median age is 18.4 years (8). National health services is managed by the Ministry of Public Health (MoPH) financed by donors and the government, and services are delivered based on two packages: Basic Packages of Health Services (BPHS) and Essential Package of Hospital Services (EPHS). These two packages were the main reforms that happened in Afghanistan's health system and had considerable effects on the health situation and indicators in Afghanistan. BPHS included the most needed services at the health posts and health center levels of the health system up to district hospitals. EPHS provides guidance to enable hospitals to function more effectively as part of the health system (9).

The burden of NCDs is escalating in Afghanistan. Based on the Afghanistan Non-Communicable Diseases and Injuries (NCDI) Poverty Commission 2018 Report, 23.6% of Disability-Adjusted Life Years (DALY) burden is attributed to cardiovascular diseases, and the next diseases with high DALY burden are neoplasms (8.9%), chronic respiratory diseases (5.2%), digestive diseases (5.1%), and diabetes mellitus (4.3%) (10). The primary studies about the burden of major NCDs are limited in Afghanistan, so the specific etiology is also unclear. However, mortality trends also show the burden of NCDs. Afghanistan Mortality Survey (AMS) 2010 revealed that about 46% of mortality in Afghanistan is mainly due to cardiovascular diseases (female 17.9% and male 14%), cancer (female 8.3% and male 7.3%), diabetes mellitus (female 2.7% and male 3.7%), respiratory disease (female 2.3% and male 1.9%), and injuries. It means that every two Afghans are dying from NCDs in Afghanistan and based on the Afghanistan NCDI poverty commission 2018 report, 56.6% of NCDs DALYs and the probability of premature mortality from target NCDs is 30% in Afghanistan (3). However, it is mentionable that the mortality data are related to the pre-COVID era, and huge shifts are expected in many countries in the share of communicable diseases and NCDs since the emergence of COVID-19. Despite its high burden, NCDs have ranked very low among government and donor priorities. The MoPH recently developed a national strategy for the prevention and control of NCDs 2015–2020 (11) and is currently working on the second strategy for 2022–2027. NCD-related health service delivery is still limited by the Afghanistan health system. The public sector lacks institutions with technical expertise for major NCDs. In this context, the private sector (not-for-profit and for-profit providers and contractors) is the prominent source of outpatient services. The for-profit sector provides mainly curative care. The national NCDs surveillance system, which is crucial for informed policy and strategy, has not been established yet. The mechanism for death registration and the qualification of the cause of death information does not exist (12). In addition, Afghanistan's health system is mainly financed by private out-of-pocket payments (77%) and development partners (13). Although several health strategies were developed for Afghanistan, there is no focus on NCDs, which is a major shortcoming as NCDs are increasing rapidly in Afghanistan.

In the first decade of the 21st century, many scientists and international organizations called for a global response to NCDs to control the global crisis (14). The initial efforts to control the NCDs pandemic started by WHO in 2010 by publishing “the Package for Essential Non-communicable (PEN) Diseases interventions for primary healthcare in low-resource settings” (15). It was followed by a high-level United Nations meeting in September 2011 that endorsed a declaration on the prevention and control of NCDs with a special focus on the challenges of developing countries (16, 17). These efforts were continued by a series of publications such as the WHO NCDs global monitoring framework; a report of WHO on the NCDs country profile; a global action plan for the prevention and control of NCDs 2013–2020; WHO regional action plan for prevention and control of NCDs; resolutions WHA63.14: restriction on marketing of foods and non-alcoholic beverages to children; global code of practice on the marketing of unhealthy food and beverages to children 2012; 2017 WHO Cancer Resolution; WHO mental health action plan 2013–2020; and Montevideo roadmap 2018–2030 on NCDs as a sustainable development priority (18). This shows the presence of many guidelines, roadmaps, and plans developed internationally which can be used by any individual country. However, in a literature review, we found two documents on controlling and preventing NCDs in Afghanistan which is as follows: the national strategy to control and prevent non-communicable diseases in Afghanistan (2015–2020) and the Afghanistan NCDI poverty commission report (2018).

International programs led to aging and longer life expectancy by addressing infectious diseases. The world bank data show that the life expectancy at birth increased from 52.5 years in 1960 to 72.7 years in 2019 globally (19). It resulted in an increased number of elderly people with NCDs, especially in low- and middle-income countries (LMICs) after a high incidence in higher-income countries. Currently, LMICs have 80% of the NCDs burden, especially in Southeast Asia and Eastern Mediterranean regions (20). NCDs also affect the economies of nations by lowering earning capacity, reducing productivity, and increasing household expenditures (21, 22).

Addressing this global challenge needs effective interventions which contribute to decreasing morbidity, premature death, and disability at the global level (20, 22). The World Health Assembly adopted a comprehensive set of indicators in 2013 as a global monitoring framework for NCDs. Since then, WHO published three sets of monitoring progress reports showing member states' status on achieving the specified targets. The reports indicate that some countries have considerable progress toward controlling and preventing NCDs. Nevertheless, many others including Afghanistan do not meet the targets. Based on the reports, Afghanistan has only conducted a national STEP survey in 2018, and there are national integrated NCD policy/strategy/action plans, smoke-free policies, and regulation on bans on advertising, promotion, and sponsorship of tobacco products. In addition, there are measures to restrict the harmful use of alcohol and policies and measures to restrict the marketing of breast-milk substitutes. Based on this progress report, the following targets are not achieved by Afghanistan: determining the national NCD targets and indicators, generating mortality data on NCDs, increasing excise taxes and prices on tobacco products, using large graphic health warnings/plain packaging on tobacco products, conducting mass media campaigns to reduce the tobacco demand, developing and implementing the strategies toward unhealthy diet reduction, addressing physical inactivity in the community, development of a guideline for the management of major NCDs, and using preventing measures for heart attacks and strokes. However, the countries which have good progress with cost-effective and feasible interventions can be a benchmark for other countries (23–25).

So, we used the NCDs progress monitor report 2020 to identify countries with a percentage of death from NCDs higher than 70% and a probability of premature mortality from target NCDs <14%. It means that these countries designed and implemented effective strategies for preventing and controlling NCDs despite their high mortality rate. We also tried to select countries from each region of the World Health Organization (African region, region of the Americas, South-East Asian region, European region, and Eastern Mediterranean region). This study aimed to identify the promising strategies to control and prevent NCDs which have already been implemented in 15 high- and middle-income countries from all over the world through a scoping review and propose the strategies which are appropriate to Afghanistan context compared to the recommended best buys interventions report of WHO (26). In this report, WHO proposed 16 interventions considered the most cost-effective and feasible for implementation in low- and middle-income countries (LMICs) with an average cost-effectiveness ratio of ≤1$100/DALY applicable in LMICs.

## 2. Methods

We conducted a scoping review from September to December 2021. Scoping review studies examine the nature, range, and extent of research activities, determine gaps in the literature, and summarize the research findings (27). We used the six-step Arksey and O'Malley protocol for this study based on the following: 1, identifying the research question; 2, searching for relevant studies; 3, selecting studies; 4, charting the data; 5, collating, summarizing, and reporting the results; and 6, consulting with stakeholders to inform or validate findings of the study (28).

We investigated the strategies to prevent and control NCDs in the selected countries. We carried out this study by searching eight international databases (PubMed through MED-LINE, Web of Science, Scopus, Science Direct, Springer, Wiley, Emerald, and ProQuest) and searching Google Scholar for literature published up to 30 November 2021. Moreover, we reviewed reports from WHO and the World Bank. We used three key terms to search the strategies (NCDs, reduction, and strategies). In addition to the key terms, we used Medical Subject Headings (MeSH) terms. Table 1 shows the search strategy in the selected international databases.

**Table 1:** Search strategy terms in selected databases.

**Database**		**Articles**
PubMed	((((exp. policy/) OR (exp. policy making/) OR (exp. legislation as Topic/) OR (exp. Government/)) OR (Government Regulation/)) OR ((prevent^*^ OR control^*^ OR program^*^ OR campaign^*^ OR policy OR strategy OR policies OR guideline^*^ OR ban OR banned OR Regulate^*^ OR law OR laws OR prohibit^*^ OR restrict^*^ OR legislate^*^ OR report^*^ OR tax OR audit^*^ OR formular^*^ OR expenditure^*^ OR spending OR label^*^ OR market^*^ OR advertise^*^ OR consultation^*^).ab,ti.)) AND (Afghanistan OR Canada OR Finland OR Iran OR Ireland OR Japan OR Malta OR “New Zealand” OR Norway OR Portugal OR Republic of Korea OR Singapore OR Spain OR Sweden OR Thailand OR “United kingdom”) AND ((“non”[tiab] AND “communicable”[tiab]) OR “non-communicable”[tiab] OR (“chronic disease”[MeSH Terms] OR (“chronic”[tiab] AND “disease”[tiab]) OR “chronic disease”[tiab]) OR “NCD”[tiab] OR (“cancer s”[tiab] OR “cancerated”[tiab] OR “canceration”[tiab] OR “cancerization”[tiab] OR “cancerized”[tiab] OR “cancerous”[tiab] OR “neoplasms”[MeSH Terms] OR “neoplasms”[tiab] OR “cancer”[tiab] OR “cancers”[tiab]) OR (“cardiovascular diseases”[MeSH Terms] OR (“cardiovascular”[tiab] AND “diseases”[tiab]) OR “cardiovascular diseases”[tiab]) OR (“hypertense”[tiab] OR “hypertension”[MeSH Terms] OR “hypertension”[tiab] OR “hypertension s”[tiab] OR “hypertensions”[tiab] OR “hypertensive”[tiab] OR “hypertensive s”[tiab] OR “hypertensives”[tiab]) OR “cvds”[tiab] OR “CVD”[tiab] OR (“stroke”[MeSH Terms] OR “stroke”[tiab] OR “strokes”[tiab] OR “stroke s”[tiab]) OR (“diabetes”[tiab] OR “diabetes mellitus”[MeSH Terms] OR (“diabetes”[tiab] AND “mellitus”[tiab]) OR “diabetes mellitus”[tiab] OR “diabetes”[tiab] OR “diabetes insipidus”[MeSH Terms] OR (“diabetes”[tiab] AND “insipidus”[tiab]) OR “diabetes insipidus”[tiab] OR “diabetic”[tiab] OR “diabetics”[tiab] OR “diabets”[tiab]) OR (“diabetes mellitus”[MeSH Terms] OR (“diabetes”[tiab] AND “mellitus”[tiab]) OR “diabetes mellitus”[tiab]) OR (“diabetes mellitus, type 2”[MeSH Terms] OR “type 2 diabetes mellitus”[tiab] OR “type 2 diabetes”[tiab]) OR ((“raise”[tiab] OR “raised”[tiab] OR “raises”[tiab] OR “raising”[tiab] OR “raisings”[tiab]) AND (“blood pressure”[MeSH Terms] OR (“blood”[tiab] AND “pressure”[tiab]) OR “blood pressure”[tiab] OR “blood pressure determination”[MeSH Terms] OR (“blood”[tiab] AND “pressure”[tiab] AND “determination”[tiab]) OR “blood pressure determination”[tiab] OR (“blood”[tiab] AND “pressure”[tiab]) OR “blood pressure”[tiab] OR “arterial pressure”[MeSH Terms] OR (“arterial”[tiab] AND “pressure”[tiab]) OR “arterial pressure”[tiab] OR (“blood”[tiab] AND “pressure”[tiab]))) OR (“hypertension”[MeSH Terms] OR “hypertension”[tiab] OR (“high”[tiab] AND “blood”[tiab] AND “pressure”[tiab]) OR “high blood pressure”[tiab]) OR ((“raise”[tiab] OR “raised”[tiab] OR “raises”[tiab] OR “raising”[tiab] OR “raisings”[tiab]) AND (“cholesterol”[MeSH Terms] OR “cholesterol”[tiab] OR “cholesterol s”[tiab] OR “cholesterol”[tiab] OR “cholesterols”[tiab])) OR (“obeses”[tiab] OR “obesity”[MeSH Terms] OR “obesity”[tiab] OR “obese”[tiab] OR “obesities”[tiab] OR “obesity s”[tiab]) OR ((“raise”[tiab] OR “raised”[tiab] OR “raises”[tiab] OR “raising”[tiab] OR “raisings”[tiab]) AND “BMI”[tiab]) OR (“overweight”[MeSH Terms] OR “overweight”[tiab] OR “overweighted”[tiab] OR “overweightness”[tiab] OR “overweight”[tiab]) OR “BMI”[tiab] OR (“exp”[tiab] AND (“alcohol drinking”[MeSH Terms] OR (“alcohol”[tiab] AND “drinking”[tiab]) OR “alcohol drinking”[tiab])) OR (“exp”[tiab] AND (“drinking behavior”[tiab] OR “drinking Behavior”[MeSH Terms] OR (“drinking”[tiab] AND “Behavior”[tiab]) OR “drinking Behavior”[tiab])) OR (“exp”[tiab] AND (“tobacco use Disorder”[MeSH Terms] OR (“tobacco”[tiab] AND “Disorder”[tiab]) OR “tobacco use Disorder”[tiab] OR (“tobacco”[tiab] AND “dependence”[tiab]) OR “tobacco dependence”[tiab])) OR “diet^*^”[tiab] OR (“diet”[MeSH Terms] OR “diet”[tiab] OR “dietary”[tiab] OR “dietaries”[tiab]) OR (“nutrition s”[tiab] OR “nutritional status”[MeSH Terms] OR (“nutritional”[tiab] AND “status”[tiab]) OR “nutritional status”[tiab] OR “nutrition”[tiab] OR “nutritional sciences”[MeSH Terms] OR (“nutritional”[tiab] AND “sciences”[tiab]) OR “nutritional sciences”[tiab] OR “nutritional”[tiab] OR “nutritional's”[tiab] OR “nutritions”[tiab] OR “nutritive”[tiab]) OR (“physical”[tiab] AND “inactivity”[tiab]) OR “physical inactivity”[tiab] OR (“tobacco use”[MeSH Terms] OR “tobacco”[tiab] OR “tobacco use”[tiab] OR (“tobacco”[tiab] AND “consumption”[tiab]) OR “tobacco consumption”[tiab]) OR “walk^*^”[tiab] OR (“junk”[tiab] AND (“food”[MeSH Terms] OR “food”[tiab])) OR (“fast foods”[MeSH Terms] OR (“fast”[tiab] AND “foods”[tiab]) OR “fast foods”[tiab] OR (“fast”[tiab] AND “food”[tiab]) OR “fast food”[tiab]) OR (“risk factors”[MeSH Terms] OR (“risk”[tiab] AND “factors”[tiab]) OR “risk factors”[tiab]) OR ((“riskiness”[tiab] OR “risky”[tiab]) AND “behavior”[tiab]) OR ((“riskiness”[tiab] OR “risky”[tiab]) AND “behavior”[tiab]) OR “physical activity”[tiab] OR “tobacco cessation”[tiab] OR “smoking cessation”[tiab])	1,108
Scopus	TITLE-ABS-KEY (“chronic disease” OR “non-communicable disease” OR “non-communicable disease” OR “non-communicable disease” OR “NCD” OR “cancer” OR “cardiovascular diseases” OR hypertension OR stroke OR “diabetes mellitus” OR “blood pressure” OR tobacco OR “physical activity” OR “physical inactivity” OR obesity) AND TITLE-ABS-KEY (afghanistan OR canada OR finland OR iran OR ireland OR japan OR malta OR “New Zealand” OR norway OR portugal OR republic AND korea OR Singapore OR spain OR sweden OR thailand OR “United kingdom”) AND TITLE-ABS-KEY (prevent^*^ OR control^*^ OR program^*^ OR campaign^*^ OR policy OR “national strategies” OR “national policies” OR guideline^*^ OR regulat^*^ OR law OR laws OR legislat^*^ OR tax) AND (LIMIT-TO (PUBSTAGE, “final”)) AND (LIMIT-TO (OA, “all”)) AND (LIMIT-TO (LANGUAGE, “English”)) AND (LIMIT-TO (DOCTYPE, “ar”) OR LIMIT-TO (DOCTYPE, “re”) OR LIMIT-TO (DOCTYPE, “ed”) OR LIMIT-TO (DOCTYPE, “cp”) OR LIMIT-TO (DOCTYPE, “le”) OR LIMIT-TO (DOCTYPE, “bk”))) (40 pages reviewed)	2,000
Web of Science	((ALL = (“chronic disease” OR “non-communicable disease” OR “non-communicable disease” OR “non-communicable disease”)) AND ALL = (prevent^*^ OR control^*^ OR program^*^ OR campaign^*^ OR policy OR “national strategies”)) and Open Access and Articles or Review Articles or Proceedings Papers (Document Types) and All Open Access (Open Access) and English (Languages) (20 pages reviewed)	50
Springer	(“Non-communicable disease” OR “non-communicable disease” OR “non-communicable disease”) AND (prevent^*^ OR control^*^) within English 1965–2021(50 pages reviewed)	1,000
Wiley	“(“Non-communicable disease” OR “non-communicable disease” OR ”non-communicable disease“)” anywhere and “(prevent^*^ OR control^*^)” anywhere (10 pages reviewed)	215
Emerald	(“Chronic disease” OR “non-communicable disease” OR “non-communicable disease” OR “non-communicable disease” OR “NCD” OR “cancer” OR “cardiovascular diseases” OR hypertension OR stroke OR “diabetes mellitus” OR “blood pressure” OR tobacco OR “physical activity” OR “physical inactivity” OR obesity) AND (afghanistan OR canada OR finland OR iran OR ireland OR japan OR malta OR “New Zealand” OR norway OR portugal OR korea OR singapore OR spain OR sweden OR thailand OR “United kingdom”) AND (prevent^*^ OR control^*^ OR program^*^ OR campaign^*^ OR policy OR “national strategies” OR “national policies” OR guideline^*^ OR regulat^*^ OR law OR laws OR legislat^*^ OR tax)	410
ProQuest	(“Non-communicable disease” OR “non-communicable disease” OR “non-communicable disease”) AND (prevent^*^ OR control^*^) (10 pages reviewed)	62
GoogleScholar		53
Final		4,898

The inclusion criteria were English documents and evidence produced up to 30 November 2021 for the control of NCDs in selected countries (Iran, Malta, Thailand, Spain, Sweden, Singapore, United Kingdom, Republic of Korea, Portugal, Norway, New Zealand, Japan, Ireland, Finland, and Canada). These countries had a percentage of death from NCDs higher than 70% and the probability of premature mortality from target NCDs <14%. It means these countries designed and implemented effective strategies for preventing and controlling NCDs despite their high mortality rate. We excluded incomplete texts, duplications, and dissertations (due to lack of access).

We used the NCDs country profile 2018 developed by WHO to find out the countries with the best policies, protocols, and measures. Importantly, these documents were used to track the progress of Member States toward achieving the global targets for NCDs to be reached by 2025. Three researchers independently extracted and created summary sheets in Microsoft Excel. The unobtainable data were recorded as missing data. One of these researchers compared the collected data after data extraction and made a single data sheet for analysis.

**Figure 1:**
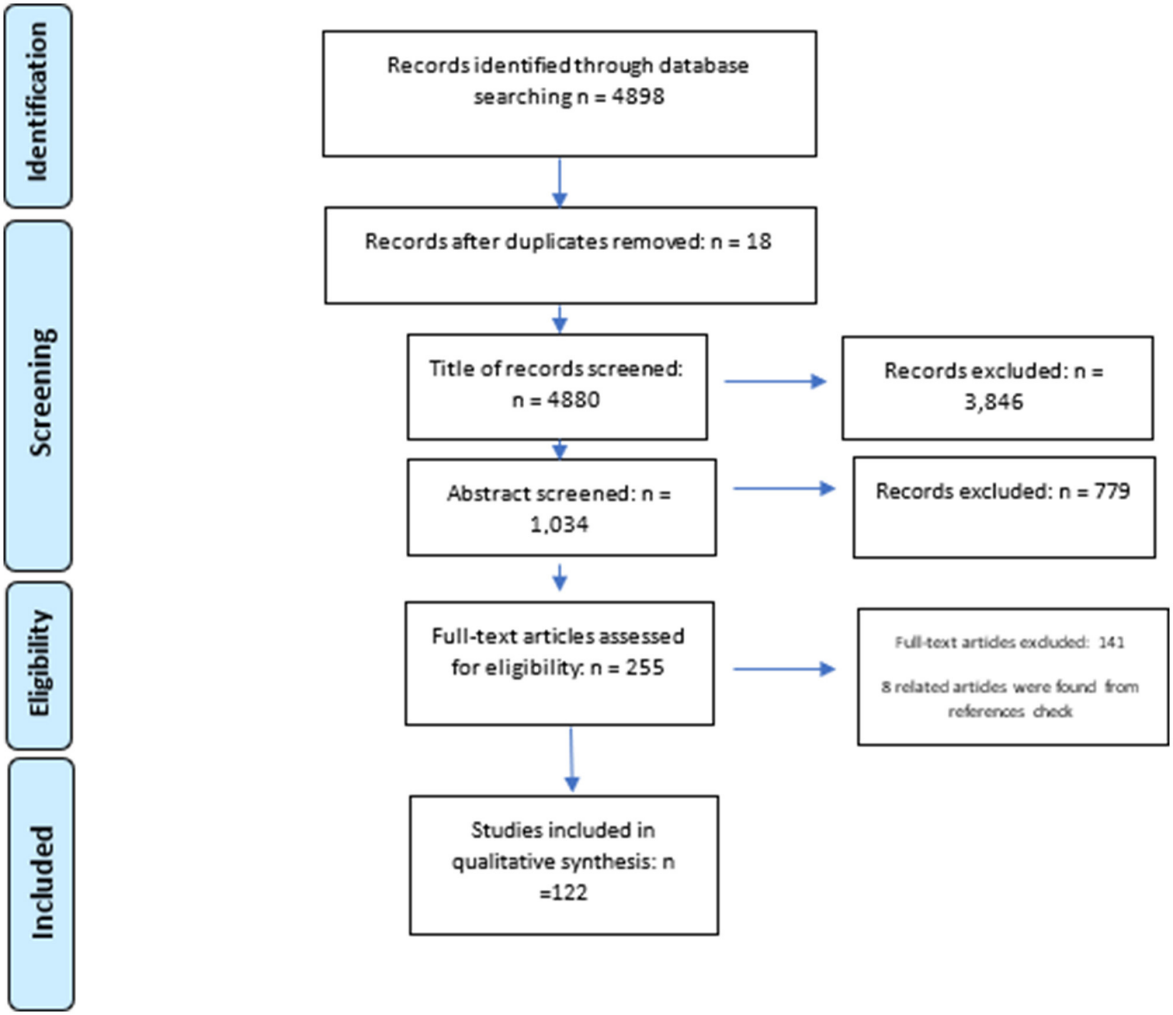
PRISMA flow diagram depicting the study selection process.

We followed the Preferred Reporting Items for Systematic review and Meta-Analysis (PRISMA) guidelines (29). The initial search resulted in 4,898 articles. We used EndNote X9 software to exclude duplications and irrelevant studies and organize the references. We removed 18 duplicated papers. After screening for relevant titles, we excluded 3,846 studies and selected 1,034 studies for abstract screening. We removed 779 articles after reviewing their abstracts. These studies did not include any strategies for preventing and controlling NCDs and their risk factors. We removed 141 additional articles after examining the full texts. Finally, we found 114 documents eligible for inclusion in this scoping review. We manually searched the reference lists of the 114 articles and added eight studies to eligible articles. Figure 1 demonstrates the search process.

The researchers designed a data-gathering form based on the study's objective. They used the content analysis method for data analysis *via* MaxQDA software. Content analysis as a research method is a systematic and objective means of describing and exploring phenomena. It is also known as a method of analyzing documents. Through content analysis, we can categorize the words and phrases into similar groups which have similar meanings and generate ideas (30).

We used the major NCDs and their risk factors as themes (cardiovascular diseases, cancer, diabetes, hypertension, tobacco use, unhealthy diets, harmful use of alcohol, and physical inactivity/obesity). We also generated a separate theme for studies that target multiple of these diseases and risk factors as integrated strategies, as most of the strategic plans of these countries addressed most/all the NCDs and their risk factors, but the published papers usually addressed only one or two of these diseases or their risk factors. Then, we extracted the strategies from the studies and categorized them under related themes.

## 3. Results

In this section, we present the characteristics of the identified studies and elaborate on the strategies under nine themes including tobacco use, healthy diet, harmful use of alcohol, physical inactivity/obesity, heart diseases, cancers, diabetes mellitus, hypertension, and integrated strategies, which address multiple of the mentioned factors.

### 3.1. Main characteristics of reviewed articles/documents

**Figure 2:**
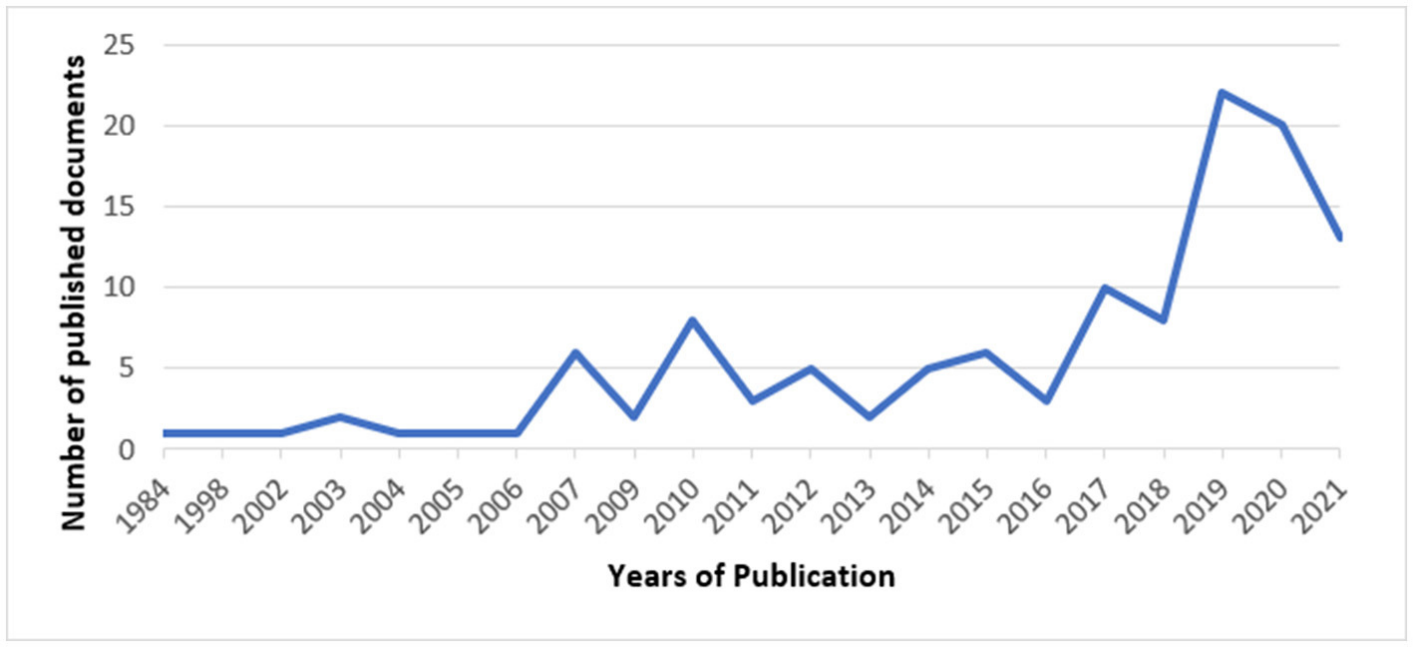
Frequency distribution of published documents on NCDs strategy per year.

A total of 122 articles/documents have been identified that were developed during 1984–2021. Almost 60% of the studies have been conducted after 2017 (Figure 2).

**Figure 3:**
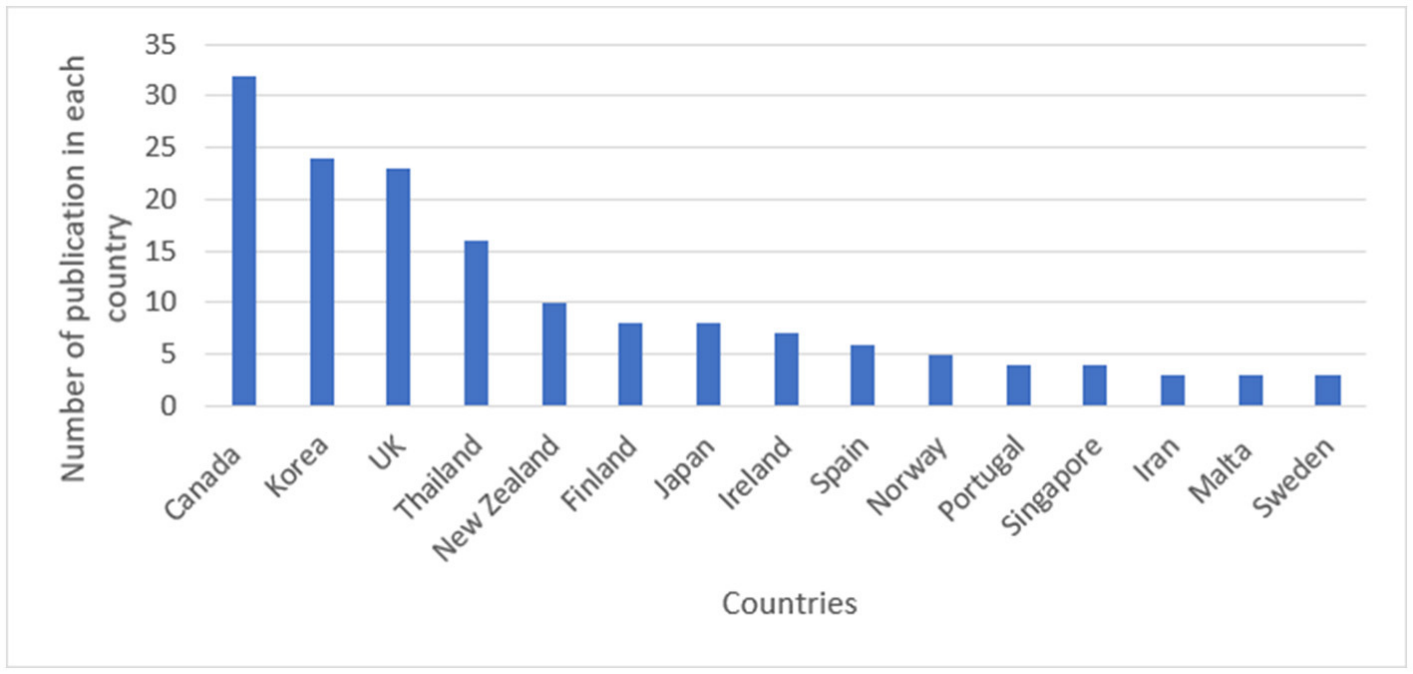
Frequency distribution of published documents on NCDs strategy per country.

Canada (26.4%), Korea (19.8%), and the United Kingdom (19%) have the most publications on the control and prevention of NCDs among the countries included in the study (Figure 3).

**Figure 4:**
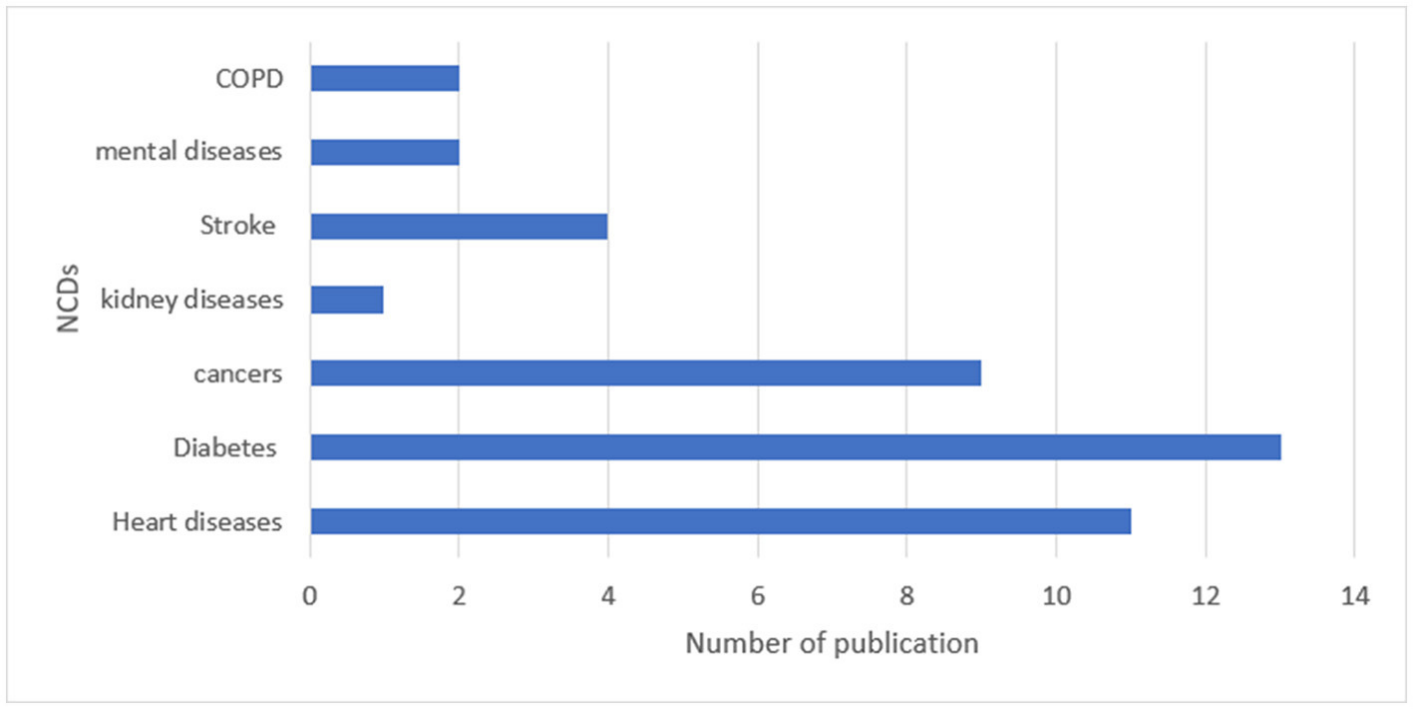
Frequency distribution of publications focused on NCDs.

**Figure 5:**
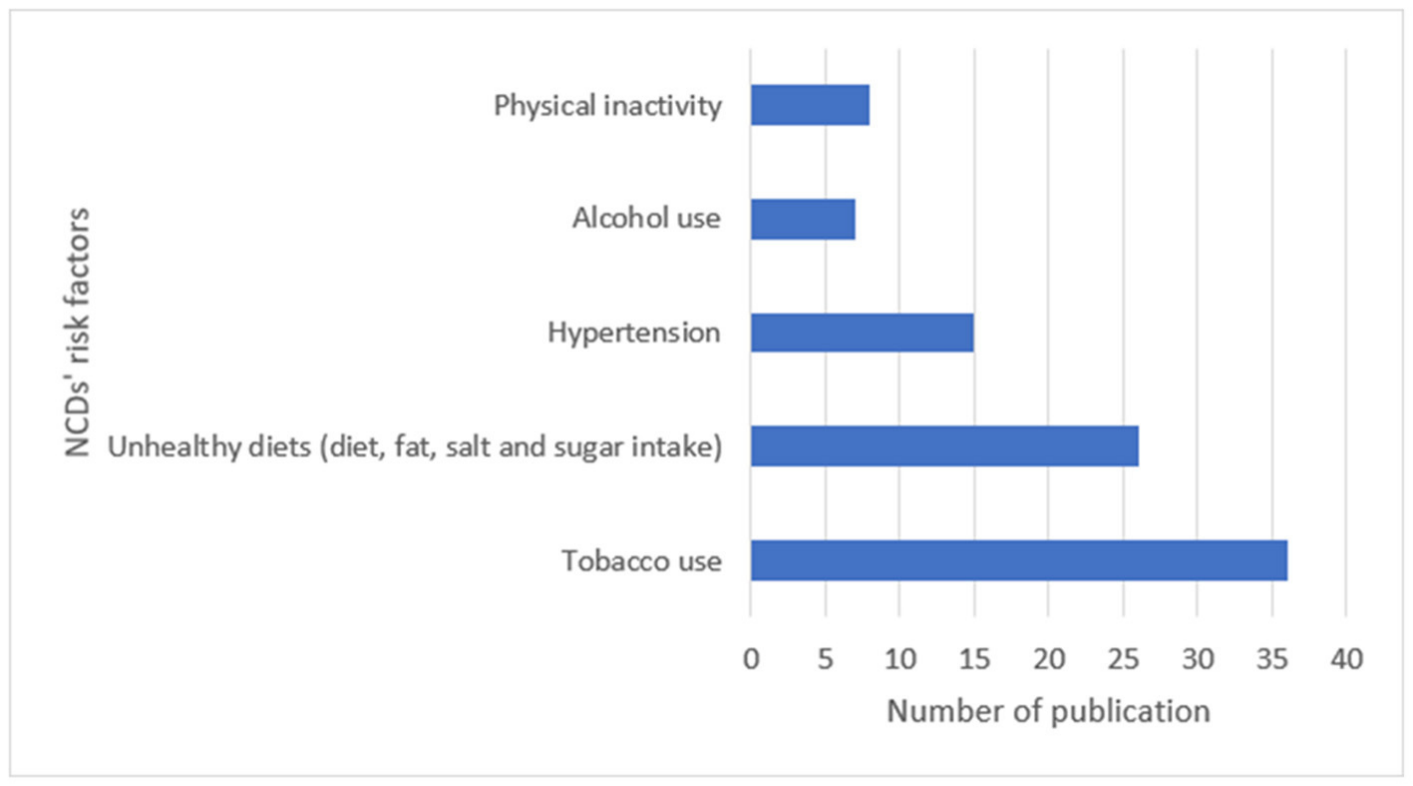
Frequency distribution of publications focused on risk factors.

Approximately 79.3% of the studies were original articles. The remaining (30.7%) were document reviews, action plans, and review articles. As Figures 4, 5 show, most of the studies were focused on how to control and prevent diabetes, heart disease, and tobacco use.

### 3.2. Strategies to prevent and control NCDs

We identified 35 strategies in this study, which we categorized under nine themes presented in the Method section. The number of strategies under each theme is presented in Table 2.

**Table 2:** Population-based strategies for the prevention and control of NCDs.

**Themes/factors**	**Strategy**	**Frequency**
Tobacco use	Smoking workplace cessation program: Restrictions and ban on smoking in public places; Development of fiscal policy on tobacco; Regulation of tobacco as a product; Government restrictions on cigarette promotion; Warning labels on packs of cigarettes; prohibiting sales of tobacco to children; Appropriate training for healthcare professionals; Public education and information campaigns; Nicotine Replacement Therapy (NRT)	10
Unhealthy diets	Development of dietary guidelines for public and voluntary programs for food manufacturers and monitoring the food industry; Regulating food and beverage advertising in social media; Food Labeling Strategy; Assignment of dietitians in primary healthcare clinics and schools; taxation on unhealthy foods; Public awareness campaigns	6
Harmful use of Alcohol	Establishing Focus Resource Center; Cancer Warning Labels on Alcohol Containers	2
Physical inactivity/obesity	Workplace and School-Based Physical Activity and change behavior Programs; Free training on physical activity for disabled or high-risk people; weight management support via the workplace; develop guidance documents on healthy eating, physical activity, and health weights for professional health staff	4
Cardiovascular diseases	Integrate heart health into the existing community health system	1
Cancer	Involving patients and family advisors in developing cancer strategic plan; develop database for cancer screening programs; National cancer screening programs	3
Diabetes	Paying for performance on the management of type 2 diabetes mellitus; Diabetic patients' empowerment for selfcare; Development of Policy to control and prevent diabetes	3
Hypertension	Development of screening program for individuals with hypertension in the community; Selfcare training sessions for adults with uncontrolled hypertension; Development of national plan for reducing sodium intake	3
Integrated strategies	Development of national strategic plan to control and prevent NCDs; Developing and passing act on providing long term health care and insurance for NCDs; development of regional action plan for NCDs by WHO	3

#### 3.2.1. Tobacco use

Smoking cessation program is the most frequent strategy applied by most countries. Workplace smoking cessation program was applied in Korea, Thailand, and the United Kingdom. A workplace smoking cessation program in Korea effectively encouraged autonomous regulation and competence for workers. This program was based on self-determination theory using individual counseling and tailored text messaging for 12 weeks (31). Restriction and ban on smoking in public places are another strategy applied by some governments and was effective in declining smoking prevalence in federal jurisdictions of Canada (32–34), the United Kingdom (35, 36), Thailand (37, 38), and Korea (39, 40). However, a study in the United Kingdom showed low levels of clinician support for recommending e-cigarettes to patients with cancer (41).

Fiscal policies are used to reduce the demand for tobacco use by increasing cigarette prices and taxes on tobacco in many countries including Thailand, Malta, Portugal, Sweden, Ireland, Canada, and the United Kingdom (36, 41–43). Korean government imposed a 10% value-added and waste disposal tax of 7 Won on 20 cigarettes during 1994–2014. This policy led to more cutbacks on smoking among lower-income smokers than among higher-income smokers in Korea (40).

Ban on all tobacco advertisements or restriction on tobacco point of sale is the demand reduction measures applied in many countries (32, 36, 43–46). Canada passed the Smoke-Free Ontario Act (SFOA) in 2006 by which restrictions were applied on tobacco point of sale in any place. The government aimed to restrict youth access and smoking in enclosed workplaces and public places (35). Japan also banned tobacco advertisements on broadcast media on TV commercials aired between 5 am and 10:54 pm based on its tobacco policies in 2005 (45). In the United Kingdom, a committee on advertising practice has produced guidelines that balance the protection of minors and the promotion of new low-risk products to consumers (46).

Warning labels on packs of cigarettes including text or graphic pictorial messages are used to decrease the prevalence of smoking in many countries. The United Kingdom also passed a regulation in 2009 to control label packaging according to the chemical of cigarettes (47). This country also passed another tobacco plain packaging legislation to reduce the brand equity and diminish the industry's long industry (48). An experimental study examined the effect of warning labels on cigarette packs in Korea between 2010 and 2012. The findings of that study support the efficacy of graphic pictorial warnings across diverse geographical and cultural contexts and support sharing health warning images across jurisdictions (49). New Zealand and Korea also used graphic pictorial warning labels (PWLs) on cigarette packs to increase non-smoking intention among adolescents (49–51). Japan also included a warning label “Be careful not to smoke too much, as smoking may be harmful to your health” on the pack of cigarettes in its tobacco policies (46).

The United Kingdom used legislation to prohibit tobacco sales to children (52) or set a committee to develop guidelines that balance the protection of minors and the promotion of new low-risk products to consumers (53). High school students were trained in a 1-h orientation program about tobacco use and the challenges of its use in a field randomized controlled trial in Iran. In addition, measures were taken to ban tobacco sales in the proximity of schools and sale to high school students (52). As part of a nationwide school-based smoking prevention program, Korea conducted a study among students aged 10–18 years in 2020. The study highlights how the school environment is associated with adolescent smoking behavior, and the effects programs and norms differ with gender. The findings of this study suggested the need to develop strategies to enhance school-based tobacco control programs and the school norm considering gender differences (54). Korea then designed a nationwide school-based smoking prevention program for students from grades 1 to 12 in 2021. This program aims to achieve a tobacco-free generation in Korea (55). In addition, through a randomized control trial study, 160 smokers from the 10th to 12th grades in Korea were included in a teen tobacco use cessation program in 2019 (56).

Providing appropriate training for health workers was another strategy for controlling tobacco use. Department of family medicine in the college of medicine at Korea Hanyang university provided a 1-year program for fourth year medical students in which they attended a role play or standardized patient's module of smoking- cessation counseling training for medical students. As a result of the program, medical students were equipped with enough knowledge to deal with a smoker willing to quit (56). Public education and information campaigns and Nicotine Replacement Therapy (NRT) are other interventions used in the European Union (35).

#### 3.2.2. Healthy diet

New Zealand developed guidelines that recommended a dietary pattern emphasizing a variety of nutritious and limiting energy-dense and nutrient-poor food products for the public (57). Some countries such as the United Kingdom, New Zealand, and Canada also designed voluntary programs for processed food manufacturers, retailers, and caterers to reduce salt intake in the general population (58, 59). Canada passed the Consumer Protection Act in 1980; and then it restricted the commercial marketing of unhealthy foods to children (60). A study in the United Kingdom assessed the adolescents' awareness of marketing for foods high in fat, salt, or sugar (HFSS), and the association between the past month's awareness and weekly HFSS food consumption. The authors found a causal relationship between marketing awareness and consumption and proposed to the United Kingdom government for further restrictions to reduce HFSS consumption (61). The government of Canada also tried to promote healthy diets and lifestyles in all child-directed activities (completely refrain from marketing products to children or to only market products that satisfy the CAI's Uniform Nutrition Criteria) (62, 63).

The front-of-pack labeling strategy was also reported in Canada and Portugal. Simplified nutrition information is an effective population-wide intervention to improve NCD outcomes. It leads to making the healthier and easier choice for consumers and encouraging manufacturers' reformulation of their food products toward offerings of higher nutritional quality (64, 65). A study in Canada shows the strength of using free sugar DV labeling to discourage the selection of products with excess free sugars (66). Boon et al. (67) reported that most Canadian consumers now use National Health Products to make informed consumer decision-making to choose healthy foods. A study in the United Kingdom showed that modest dietary improvements in the United Kingdom could avert approximately 12,000 CVD deaths annually and more substantial improvements could avert about 30,000 CVD deaths annually (68). Assignment of dietitians in primary healthcare clinics and schools is one of the strategies to promote a healthy diet in the community. Another study in New Zealand investigated the effectiveness of dietetic intervention in primary healthcare on health and wider economic outcomes. The study concluded that dietitians in primary healthcare can improve patients' health and quality of life. Increasing the number of dietitians working in primary healthcare has the potential to make quality nutrition care accessible and affordable for more New Zealanders (69). Face-to-face lifestyle counseling sessions were provided to the adult in the families in a study in European Countries during the first year of intervention; while during the second year, the intervention was implemented *via* personalized SMS messages. The program resulted in the improvement of certain lifestyle behaviors in parents from high-risk families (70). The Korea Institute of Child Care and Education designed a similar program in Korea. A 10-week nutrition intervention was performed by a team of nutrition professionals with the cooperation of 14 class teachers at the seven daycare centers and kindergartens in 2016. This program improved young children's eating behaviors and nutrition status (71). Taxation of sugary drinks and education and awareness of consumers, industry, and health professionals are other strategies suggested in some studies (59, 60, 62).

#### 3.2.3. Harmful use of alcohol

Casswell et al. (72) in 2012 designed an instrument to assess the impact of alcohol control policies. He and his team then conducted the International Alcohol Control (IAC) study that assessed the impact of alcohol control policies on consumption and policy-related behaviors in New Zealand. The study contributed to the debate on appropriate policy responses to reduce alcohol-related harms (73). Canada established the Focus Resource Center from 2002 to 2008, which provided a wide range of services, including networking, training, consultation, and information dissemination, to 22 community programs throughout Ontario province. This intervention increased the capacity of health promoters through training, organizing, and collective efforts, and providing common services to control the harmful use of alcohol (74). Another initiative used in Canada is Cancer Warning Labels on Alcohol Containers. Placing cancer warnings on alcohol containers could make a pivotal difference in motivating both drinkers to consume less and regulators to introduce more effective policies to reduce the serious harms of alcohol consumption (75). A review study in 2017 found that screening and appropriate interventions for hazardous alcohol use and use disorders could lower blood pressure levels, but there is a lack and gap in implementing these measures in European primary healthcare. They provided some recommendations in this regard and proposed that implementing these recommendations should be in controlled settings with evaluation (76).

#### 3.2.4. Physical inactivity/obesity

Many countries designed different interventions to promote physical activity and prevent obesity among the population. A 1-year badminton and table tennis program was designed in Korea for 120 high school students in 2018 (35 min/day, three times a week). School-based physical activity programs can positively affect adolescents' physical fitness which is expected to assist health and education professionals to plan or make decisions on strategies that can promote physical activities in schools (77). A cluster randomized controlled trial also targeted junior high school students in Thailand to modify their health beliefs toward obesity and physical activity. They first trained the students on obesity and its causes in children using cartoon animation, then they informed the students on the perceived benefit of the prevention of obesity, and finally, the students were trained on cues to action for the prevention of obesity. The researcher used a family-centered approach by visiting students' homes to stimulate awareness and change behavior based on family support (78).

Countries such as Canada, Iran, and Thailand provided free physical activity training for disabled or high-risk people. Thailand designed a lifestyle modification program for rural obese women for 16 weeks. It included nutritional counseling, health education, and exercise training, leading to a loss of weight of 10.2% from baseline (79). A weight reduction program was also designed in Thailand using Facebook in 2016. They posted exercise videos from an exercise expert over a Facebook private group and assessed the program *via* daily weight reporting and monthly personalized feedback. The result of the study proves that health education and support services through Facebook can be effectively used for weight reduction among students at a public university in Thailand (80). Iran also designed a randomized control trial including theory-based training intervention on physical activity and blood pressure in hypertensive patients for 1 year (81). Canada also provides financial support for disabled athletes nationally. This promoted physical activity and recreational sports through bilateral agreements with territories and provinces (82). In some countries such as the United Kingdom, organizations also provided weight management support *via* the workplace for their staff through a partnership scheme with Slimming World Organization for a 12-week duration. The program was especially effective for people with high BMIs (83, 84). In a loyalty scheme-based program in Ireland, rewards are provided to public sector employees to increase their physical activities (85). A scale-up and scale-out of gender-sensitized weight management and healthy living program in European countries such as Canada, New Zealand, and England recruited men aged 35–65 years who were at increased risk of future ill health because of their body size (BMI > 28 kg/m^2^). The participants in this program were provided a 12-week group of 90 min sessions in Football Fans in Training (FFIT) (86). Canada also provided guidance documents about healthy eating, physical activity, and healthy weights for professional staff employed by local health boards in 2010 (87) (Table 2).

#### 3.2.5. Heart diseases

Heart Health Action Program was designed and implemented in Canada from 1990 to 1996 to integrate heart health into the existing community health system by the health promotion branch of the Ontario ministry of health (74). A study in the United Kingdom assessed the General Practitioners' (GPs) familiarity with and use of cardiovascular clinical prediction rules in 2014. Their target was a network of doctors in the United Kingdom with more than 238,000 members. They found that the GPs' knowledge and use of the CardioPulmonary Resuscitations (CPRs) technique changed substantially. They also recommended integrating CPRs into guidelines and practice software which might increase familiarity and use (88).

#### 3.2.6. Cancers

Countries used different strategies to prevent and control cancer incidence among the population. Cancer Care Ontario developed its Cancer Plan IV in 2015–2019 while cancerous patients and family advisors were involved in planning. The engagement intended to ensure that the plan would be meaningful to patients and families and help to improve the quality of care and patient experience (88). Sweden and Finland developed a web-based application to provide access to a standardized, evidence-based database for Nordic Cancer screening. This database had performance and outcome indicators of cancer screening based on the up-to-date Nordic Cancer screening register data in 1980 which is used for quality assurance and improvement of cancer screening programs (89). The cervical cancer screening program was another strategy implemented in countries such as Korea, Thailand, and Norway (90, 91).

#### 3.2.7. Diabetes mellitus

Ireland used a pay-for-performance system to manage type 2 diabetes mellitus. O'Connor et al. (92) showed substantial improvement in the process and quality of care in managing patients with type 2 diabetes mellitus in Ireland. Providing education to diabetic patients for self-management is also used in Korea and Thailand through face-to-face training sessions, text content, quizzes and video links, medication reminder, and emergency calls (93, 94). The Singapore Ministry of Health declared War on Diabetes (WoD) to rally a whole national effort to reduce the diabetes burden in the country in 2016. They developed a national policy to control and prevent diabetes. In 2021, a study showed that WoD policy generated a sense of unity and purpose across most policy actors. Policy actors were cognizant of the thrust of the policy and have begun to make shifts to align their interests with the government policy (95).

#### 3.2.8. Hypertension

Self-care training sessions for adults with uncontrolled hypertension are a strategy used to control blood hypertension in Canada and Thailand. In total, 128 participants aged 35–74 years with hypertension received a weekly email newsletter regarding hypertension management in a user-and-expert-driven web-based hypertension program during 2012–2014 in Canada. This program allowed participants to choose their intervention goals such as self-care tips for exercise or a heart-healthy diet (96). Self-management programs were implemented for Thais with essential hypertension in 2009 and 2018–2019. The program included group discussions to assess knowledge and health beliefs regarding hypertension, sodium restriction strategies, developing a self-care behavior booklet, and coaching family caregivers concerning monitoring older adults with uncontrolled hypertension (97, 98). Japan used a screening program for the detection of hypertension through an annual health checkup of 30 years and older residents during 1963–1966. This intervention led to a substantial decline in the prevalence of severe hypertension and a decline in stroke incidence in the 1960s (99). Developing a national plan to reduce dietary sodium is another strategy used in Canada (100) (Table 2).

#### 3.2.9. Integrated strategies

The identified integrated strategies are developing a national strategic plan for NCDs, developing and passing an act on providing long-term care and insurance, and developing a regional action plan for NCDs by international agencies. National strategic plans of Korea, Iran, Thailand, and Malta were reviewed in this study, designed for 6–10 years of implementation (101–105). Japan passed different acts to address the NCDs throughout the country including Health and medical service Act (1983), the long-term care insurance Act (1997), and the health promotion Act (2004) which all aimed at primary prevention as the main strategy for NCDs control in Japan (106). Some international agencies such as World Health Organization also developed a regional action plan for preventing and controlling non-communicable diseases in terms of intervention for population and individual levels for all member states (107). In a Chronic Diseases Self-Management Program (CDSMP) in Canada, patients aged between 18 and 80 years were enrolled. This standardized program proposed weekly 2.5 h meetings of 10–12 people for 6 weeks, facilitated by two peer leaders with chronic diseases. During the meetings, several issues are discussed: techniques to deal with problems such as frustration, fatigue, pain, and isolation; appropriate exercise for maintaining and improving strength, flexibility, and endurance; and appropriate use of medication and communicating effectively with family, friends, and health professionals (108) (Table 2).

## 4. Discussion

We conduct this study to identify strategies used for the prevention and control of non-communicable diseases in 15 countries (Canada, Finland, Iran, Ireland, Japan, Malta, New Zealand, Norway, Portugal, Republic of Korea, Singapore, Spain, Sweden, Thailand, and the United Kingdom). These countries had a percentage of death from NCDs higher than 70% and the probability of premature mortality from target NCDs <14%. It means that these countries designed and implemented effective strategies for preventing and controlling NCDs despite their high mortality rate. We explored the strategies for the four main risk factors of NCDs (tobacco use, unhealthy diets, physical inactivity, and harmful consumption of alcohol) and the four major non-communicable diseases (heart diseases, cancers, diabetes, and chronic obstructive pulmonary diseases) (14). There were more publications after 2017, the main reason for this could be including the non-communicable diseases in the Sustainable Development Agenda and assigning one target to these diseases (target 3.4), which has been followed by several publications, especially by WHO. Multi-sectoral and population-based preventive measures are advised in many literature studies including regulation, policy change, and market control toward NCDs prevention and control (14).

We identified 35 strategies in this study, among which the highest belongs to tobacco use (10 strategies) followed by unhealthy diet (6 strategies). We compared the identified strategies with the Best Buys intervention of WHO and proposed the cost-effective ones for Afghanistan that have not been addressed in the Afghanistan NCD strategy. Afghanistan Ministry of Public Health has only endorsed one strategy for NCDs for the duration of 2015–2020 (Afghanistan MoPH-NCD strategy) and we use it as a reference in this study (12).

Several articles have shown that tobacco use and an unhealthy diet are linked to increasing NCDs (109–112). Tobacco use causes at least 16 different types of cancer. Thakur Jha et al. showed that smoking would lead to around 930,000 adult deaths in India alone in 2011. Reducing tobacco use is one of the best strategies for preventing NCDs, along with reducing the harmful use of alcohol and promoting a healthy diet and physical activity (113). Tobacco control has an important effect on NCDs burden, and the related programs have high feasibility toward the whole population with the most benefit for the poor resulting in reducing inequities. Therefore, the priority of all countries in the world should be achieving the global goal by 2040 of a world essentially free from tobacco where <5% of the population uses tobacco (14). Different strategies were introduced in various articles to control tobacco use (Table 2), of which, the smoking cessation program; fiscal policy; and restriction and ban on smoking in public places were the most common ones. In the Afghanistan MoPH NCD strategy, there was no intervention addressing smoking in the general population. Comparing our identified strategies with the “best buys interventions,” the cost-effective interventions that Afghanistan can use to control and prevent smoking are restrictions and ban on smoking in public places, development of fiscal policy on tobacco, regulation of tobacco as a product if Afghanistan produces tobacco, increasing government restrictions on cigarette promotion, use warning labels on packs of cigarettes, prohibiting sales of tobacco to children, and conducting public education and information campaigns. A study using the data from the 2015 Afghanistan Demographic and Health Survey of about 40,000 participants showed that smoking cigarettes was the most prevalent form of tobacco use among men (21.9%, 95% CI 21.2–22.7), and the prevalence rates were lower for women. The study also showed an inverse association between education and tobacco use and a positive association between occupation (agriculture and skilled and unskilled manual labor occupations) and smoking (114). Afghanistan MoPH introduced earmarked taxes for health in Afghanistan including the tax on tobacco, vehicle, and fuel (115). But the recommended strategies have not been implemented yet due to a lack of good coordination between different ministries including the Ministry of Finance, Ministry of Interior Affairs, and MoPH. However, due to non-coordinated activities, MPOWER policies led to the enforcement of smoke-free public places. In addition, the government promotes direct and indirect bans on tobacco advertising, but still, the country does not have a national anti-tobacco media campaign to alert the public about the dangers of tobacco. Health warning labels are also in the text-only form on tobacco product packaging (114). So, the most important interventions we recommend for Afghanistan are enforcing taxes on tobacco products, conducting media campaigns to alert the public about the tobacco dangers, and adding pictorial warning labels on the packaging of cigarettes.

According to WHO, one of the important risk factors for a range of chronic diseases is an unhealthy diet (116). An unhealthy diet is connected to four of the world's top 10 risk factors causing death (Hypertension, high blood glucose, overweight and obesity, and high cholesterol) (117). Hence, evidence-based nutrition interventions should be a global health priority (118). Several strategies and programs were discussed in the reviewed articles in this study. The most frequent stricter food policies include feasible reductions in saturated fats, industrial trans fats, salt consumption, restriction on commercial marketing of unhealthy foods to children, taxation of sugary drinks, Children's Food and Beverage Advertising Initiative, Nutrition Labeling Regulation, and Food marketing policies. There is no intervention in the Afghanistan NCD strategy for controlling unhealthy diets. Compared to “best buys interventions,” we recommend the development of dietary guidelines especially to reduce salt intake for public institutions such as hospitals, schools, workplaces, and nursing homes; regulating food and beverage advertising in social media; taxation on unhealthy foods especially sweetened beverages; a public awareness campaign about healthy diet focusing on reducing salt intake through a behavior change communication and mass media campaign; and food labeling strategy by a focus on reducing salt intake through the implementation of front-of-pack labeling. A review study was conducted for mapping the current nutrition policies and capacity development initiatives in Afghanistan. The study found that limited policy provisions are available to address nutrition issues due to the rising burden of non-communicable diseases, urbanization, and changing dietary patterns (119). So, the provision of policies to address the mentioned challenges in Afghanistan should be mandatory for the Ministry of Public Health.

Afghanistan is one of the countries in the world where alcohol drinking at any age is illegal for all citizens. If the locals violate the law, they will be punished under Sharia law. The total alcohol consumption per capita is 0.2 liters of pure alcohol in 2018 in Afghanistan for people 15+ years of age, compared to the average of 6.2 liters worldwide (120). So, considering the current Afghanistan context for alcohol use, we do not provide any recommendations here.

A national survey in 2018 in Afghanistan showed that the prevalence of overweight is 25.5% and the prevalence of obesity is 17.2% in Afghanistan, and these are positively associated with ages 30–44 and 45–69 years, hypertension, and type 2 diabetes. High physical activity was negatively associated with overweight/obesity in Afghanistan (121). In this review, we highlighted four strategies to promote physical activity and control overweight/obesity, but comparing with “the best buys interventions,” we recommend the following strategies to be implemented in Afghanistan: implement a community-wide mass media campaign to educate and raise awareness to the public about physical activity aimed at supporting behavioral change of physical activity levels. In addition, integrating physical activity counseling and referral into primary healthcare services, developing guidance documents on healthy eating, physical activity, and health weights for professional health staff, and workplace and school-based physical activity programs are other implementable interventions in Afghanistan.

A study conducted in northern Afghanistan in 2018 showed that the prevalence of coronary artery disease among patients was 17.4%, with female patients having a higher prevalence rate than male patients (20.1 vs. 14.9%, *P* = 0.02) (122). In addition, a systematic review and meta-analysis study in 2021 showed that the prevalence of diabetes in Afghanistan is 12.13% and the main risk factors include increasing age, obesity, and hypertension (123). While there are some services and medicines in BPHS to control and treat heart diseases and diabetes, no intervention was mentioned in the AFG NCD strategy to control and treat these diseases. So, comparing with “the best buys intervention,” we recommend development guidelines for self-care especially for managing heart diseases and diabetes, integrating heart health into the existing community health system and providing consultation to individuals who have had a heart attack or stroke and to persons with moderate to high risk (≥20%) of a fatal and non-fatal cardiovascular event in the next 10 years.

The Afghanistan Mortality Survey in 2010 shows that 8.3% of female and 7.3% of male patients die due to cancer in Afghanistan. However, the only specific intervention to address cancer in the Afghanistan NCD strategy is the establishment of the National Cancer Control Program lacking any specific detailed intervention. Considering our findings in this review, we recommend to the Afghanistan MoPH that despite establishing a national cancer screening program especially cervical cancer among women, they can start the vaccination against human papillomavirus (2 doses) for girls aged 9–13 years.

The fourth objective of WHO's Global Action Plan for Prevention and Control of NCDs 2013–2020 is “*strengthen and orient health systems to address the prevention and control of non-communicable diseases and the underlying social determinants through people-centered primary health care and universal coverage*.” The enabling actions to reach this objective are focusing on promoting a referral system, viable health financing mechanism, enhancing preventive care at the primary level, improving the availability of affordable basic techniques and essential medicines, training the health workforce, and strengthening the capacity of the health system, particularly in the primary care level, and developing and implementing a palliative care policy (124). Considering the findings of this review, we recommend conducting a situation analysis of the NCDs management system in Afghanistan and developing a national NCDs strategy based on the six building blocks of WHO.

A limitation of our study was identifying countries with a similar context to Afghanistan which has low premature mortality rates. Most low-income countries face a dual burden of diseases (communicable and non-communicable) and have a high rate of premature mortality, which means they are not successful in controlling and preventing NCDs. So, we had to select high- and middle-income countries to explore the strategies.

## 5. Conclusion

This study highlighted the strategies to control and prevent non-communicable diseases which have already been implemented in countries with low levels of premature mortality rates. The findings of this review can be used by the Afghanistan Ministry of Public Health, the WHO Afghanistan country office, and other involved stakeholders to design and implement strategies to control and prevent NCDs in Afghanistan. The political declaration of the high-level meeting of the general assembly on the prevention and control of non-communicable diseases in 2011 highlighted the need for a whole-of-government and a whole-of-society response. It acknowledged the importance and need for a multisectoral approach and including health at all government levels to address NCDs risk factors and consider determinants of health in all programs (125). So, international organizations such as the World Health Organization, United Nations Agencies, the World Bank, and other involved communities should invest in strengthening good health governance in Afghanistan. The Afghan government should focus and invest in promoting health literacy among the public and self-care toward controlling and preventing NCDs. The proposed interventions and strategies in this study are cross-sectional approaches and need good collaboration between multiple sectors.

## Author contributions

NN, MA, AM, and PI designed the research, conducted it, and wrote the paper. NN, MA, and PI extracted the data. AM had primary responsibility for the final content. NS reviewed the article and provided technical input to enrich the paper and also edited the language of the manuscript. All authors read and approved the final manuscript.
