# Supplementary material for: Strategies to tackle non-communicable diseases in Afghanistan: A scoping review
Source: Front Public Health. 2023 Feb 22;11:982416. doi: 10.3389/fpubh.2023.982416 (PMC9992526; doi:10.3389/fpubh.2023.982416)
Supplement: Supplementary file 1 [file Table_1.DOCX]

**Supplementary Data:** Summary of reviewed studies

| first Author | Year | Type of manuscript | Country | Documents/ Program Name | Effect of the program/ Documents |
| --- | --- | --- | --- | --- | --- |
| Lim | 2019 | Original research | Korea | Workplace Smoking Cessation Program based on Self-determination Theory Using Individual Counseling and Tailored Text Messaging | This program is effective in encouraging autonomous regulation and competence for workers. |
| Kang | 2020 | Original research | Korea | Korea Youth Risk Behavior Web-based Survey (KYRBS) | the presence of cohort effects in the reduction of adolescent smoking. The cohort effect was induced by smoke-free legislation. |
| Lawrence | 2019 | Original research | New Zealand | food standards program | The program’s structure and processes produce food standards outcomes that perform well in protecting public health from risks associated with nutrient intake excess or inadequacy. opportunities to set food standards to help protect public health against dietary risks are identified. |
| Labonte | 2019 | Original research | Canada | Traffic light labelling | Traffic light labelling (if used to avoid red lights when possible) could be an effective population-wide intervention to improve NCD outcomes. |
| Magnusson | 2015 | Original research | New Zealand, UK | salt reduction programs | Voluntary efforts to achieve a national salt reduction target of 6 g/day have so far proved unsuccessful in the UK, while in Australia, the Food and Health Dialogue remains unlinked from any national target. |
| Lee | 2021 | Original research | Korea | School-Based Physical Activity Programs | school-based physical activity programs can have a positive effect on the health-related physical fitness of adolescents. These results are expected to assist health and education professionals to plan or make decisions on strategies that can promote physical activities in schools. |
| Kent | 2011 | Original research | Canada | Food marketing policies | The Quebec advertising ban does not appear to be limiting the amount of food/beverage advertising seen by children aged 10 – 12. However, food categories and marketing techniques used differ in the preferred viewing of French Quebec children. |
| O'Connor | 2020 | Original research | Ireland | paying for performance’ on the management of type 2 diabetes mellitus | The ‘cycle of care ‘demonstrated much improved rates of recording of clinical and biochemical parameters, and improved achievement of targets in total cholesterol and blood pressure, but not glycosylated hemoglobin. Results demonstrate substantial improvements in the processes and quality of care in the management of patients with type 2 diabetes mellitus |
| Vardavas | 2020 | Original research | Spain | the European Tobacco Products Directive (TPD) | As the tobacco control regulatory environment of the EU develops, it is important to continue to monitor transitions between types of products, as well as trends in cessation. |
| Goiana-da-Silva | 2019 | Original research | Portugal | food industry co-regulation | The co-regulation agreement could save lives and reduce the risk of premature death in Portugal. Nevertheless, the projected impact on mortality was insufficient to meet international targets. |
| Hoekstra | 2019 | Original research | Canada | National approaches to promote sports and physical activity in adults with disabilities | The Canadian government promotes recreational sports in disabled populations by supporting programs via bilateral agreements with provinces and territories. |
| Kates | 2007 | Reviewing article | Canada | chronic disease management models for depression in primary care | There is conclusive evidence for the benefits of changing systems of care delivery to support the more effective management of depression in primary care. improved outcomes in terms of symptom reduction, relapse prevention, functioning in the community, adherence to treatment, community and workplace involvement, and satisfaction with care received. |
| Boon | 2007 | Original research | Canada | natural health product (NHP) regulations | The label requirements of the new NHP regulations were generally viewed positively by the consumers who participated in this study. Practice implications: The additional risk information may generate more NHP-related questions for health care practitioners especially with respect to possible interactions between NHPs and conventional medicines. |
| Khumros | 2019 | Original research | Thailand | modified health belief model intervention arm (HBMIA) | A modified HBM-based intervention to reduce BMI for age is effective in overweight junior high school students. |
| Labonté | 2017 | Original research | Canada | legislation to restrict the commercial marketing of unhealthy foods and beverages to children. | The discrepancies between nutrient profile models highlight the importance for policy makers to carefully evaluate the characteristics underlying such models when trying to identify a suitable model to underpin regulations restricting the marketing of unhealthy foods to children. |
| O'Flaherty | 2012 | Original research | UK, Ireland | cardiovascular mortality reductions with stricter food policies | modest dietary improvements in the UK could avert approximately 12 000 annual deaths from CVD by 2015. However, more substantial improvements could avert about 30 000 CVD deaths annually. |
| Hammond | 2019 | Original research | Korea | health warning policies on cigarette packages. | graphic pictorial warnings across diverse geographic and cultural contexts and support sharing health warning images across jurisdictions are effective. |
| Lidón-Moyano | 2018 | Original research | Malta, Norway, Portugal, Spain, Sweden, Finland, Ireland, UK | tobacco products regulations | support for the studied tobacco products regulations were positively related with European tobacco control levels of implementation both at the ecological and individual level. |
| Li | 2021 | Original research | Finland | Knowledge sharing in online smoking cessation communities | structural capital (social ties) and relational capital (reciprocity) are important motivators behind knowledge sharing in smoking cessation online health communities, and the authors found a moderating effect of the stage in quitting on the antecedents’ relationship with knowledge sharing in these OHCs. |
| Junnual | 2019 | Original research | Thailand | smoking cessation program using both the information-motivation-behavioral skills | This program increased self-esteem, attitude toward smoking, perceived control over smoking and decreased smoking per day among male high school students. |
| Ban | 2020 | Original research | UK | GPs’ familiarity with and use of cardiovascular clinical prediction rules | GPs’ familiarity and use of cardiovascular CPRs changed substantially. Integrating CPRs into guidelines and practice software might increase familiarity and use. |
| Calnan | 1984 | Reviewing article | UK | The policy of the United Kingdom government towards the control of smoking | The evidence describing various aspects of the policy-making processes suggests that the introduction of direct legislation to control tobacco production or promotion in the UK appears to be unlikely until various barriers are removed. |
| Harrington | 2021 | Original research | UK | family-based interactive lifestyle intervention | Reporting on the intervention development process, which includes stakeholder input, could yield a flexible approach for use in this emerging ‘at risk’ groups and their families. |
| Hudon | 2016 | Original research | Canada | Stanford Chronic Disease Self-Management Program (CDSMP) | The CDSMP could therefore be considered as a self-management support option for this vulnerable clientele, while taking measures to avoid too much heterogeneity among participants to improve completion rates. |
| Hoek | 2010 | Original research | New Zealand | pictorial health warnings on tobacco packaging | despite tobacco companies’ opposition to pictorial health warnings and the resource constraints facing health authorities, the implementation process was generally robust and successful. |
| Howatson | 2015 | Original research | New Zealand | effectiveness of dietetic intervention in primary health care on health and wider economic outcomes | Dietitians in primary health care can improve patients’ health and quality of life. Increasing the number of dietitians working in primary health care has the potential to make quality nutrition care accessible and affordable. |
| MacKenzie | 2004 | Reviewing article | Thailand | transnational tobacco companies and ingredients disclosure | The evidence presented highlights the importance of ingredients regulation and demonstrates how health policy can be transformed during its implementation. |
| Hwang | 2021 | Original research | Korea | Pictorial Warning Labels | Harm perception evoked by PWLs predicts a higher non-smoking intention among adolescents. In introducing PWLs, it is important to select those that can arouse sufficient awareness to increase their effectiveness. |
| Lee | 2019 | Original research | Korea | Effect of diabetes education through pattern management on self-care and self-efficacy in patients with type 2 diabetes. | Diabetes education by PM using CGMS result analysis improved life habits with a positive influence on self-care behaviors and self-care for diabetes management. |
| Hwang | 2020 | Original research | Korea | School-Based Smoking Prevention Education | Experience of smoking prevention education within a year was significantly associated with exposure to anti-tobacco media messages. |
| Kim | 2021 | research article | Korea | Nationwide School-Based Smoking Prevention Program | This study contributes to the improvement of SSPP by generating strategies based on the exploration of SSPP implementation using the experiences of teachers involved |
| Kim | 2020 | Original research | Korea | Nationwide School-Based Smoking Prevention Program | The school environment is associated with adolescent smoking behavior, and the effects of programs and norms are different by gender. |
| Hunter | 2018 | Original research | Ireland | a loyalty scheme based intervention involving rewards for increasing physical activity in public sector employees | Although the intervention successfully altered several hypothesized mediating constructs it did not translate into long-term behavior change. |
| Lim | 2020 | Original research | Korea | the Korean Community-based NCDs Prevention and Control Program (KCNPC) | patients who registered with the KCNPC program showed a reduced mortality risk compared to the control group. Lower risks of hospitalization due to complications and lower proportional risk of hospitalization were also seen among patients registered in the program. |
| Nikolaou | 2019 | Original research | UK, Finland, Singapore, New Zealand | usage of lifestyle apps | Young people are commonly, and consistently across 6 countries, concerned about weight gain and obesity and would welcome evidence-based mHealth programs, provided the views of young people themselves are incorporated in the program content. |
| Manios | 2020 | Original research | Finland, Spain | Feel4Diabetes intervention (Family- and Community-Based Intervention) | the first year of the Feel4Diabetes intervention resulted in the improvement of certain lifestyle behaviors in parents from high-risk families. |
| Kim | 2019 | Original research | Korea | the US National Aeronautics and Space Administration (NASA) Mission X (MX) Program | the program improved the eating behaviors and nutrition status of young children. |
| Byun | 2021 | Original research | Korea | Community-Based Intervention Programs for Improving Treatment Compliance of Patients with Chronic Diseases | all participants in this survey seemed to view patients’ preventive strategy as being important given clinical features of hypertension and diabetes. For sustainable management of hypertension and diabetes within regional communities, not only service provision through selection and continuous work of capable staff, but also the appropriateness of health education about health-related behavior that can improve a patient’s satisfaction and treatment adherence are important. |
| Hunt | 2020 | Original research | Canada, New Zealand, UK | The Wider Implementation of Football Fans in Training (FFIT) | public health interventions can appeal to men and support them in sustainable lifestyle change |
| Jolemore | 2006 | Original research | Canada | APTS's "To Be Tobacco Free" program | The success of the Capital approach to tobacco treatment and cessation is evident. "To Be Tobacco Free" continues to support community members who are ready to address the harms associated with tobacco dependence whether they are just getting started in their efforts to live tobacco free or successfully keeping it going |
| Casswell | 2012 | Original research | UK, Thailand, Korea, and New Zealand | Evaluating the Impact of Alcohol Policies | In a policy arena in which the interest groups and stakeholders have different perceptions of appropriate policy responses to alcohol-related harm, a robust methodology to assess the impact of policy will contribute to the debate |
| Khayyati | 2019 | Letter to the Editor | Iran | community and school-based intervention to control and prevent of tobacco use in adolescents | intervention programs are effective in preventing tobacco use in nonsmokers than those who already smoke. Involving teachers in policies and encouraging participation and cooperation among different authorities of community contribute to the control and prevention of to-bacco use. |
| Casswell | 2014 | Original research | New Zealand | the International Alcohol Control | Heavy drinkers were more likely to buy cheaper alcohol and purchase at later times; policy issues under discussion in many settings. This analysis suggests the IAC study that has the potential to provide data to contribute to the debate on appropriate policy responses to reduce alcohol-related harm. |
| Motlagh | 2017 | Original research | Iran | theory-based training intervention on physical activity and blood pressure in hypertensive patients | a three-month Theory-based training intervention used was effective in increasing PA and reducing 24 hour ABP. |
| Yu | 2019 | Original research | Korea | Project EX: a smoking intervention pilot program | Project EX is a plausible program to implement among Korean adolescents. |
| Liu | 2018 | Original research | Canada | user-and expert-driven web-based hypertension programs | It may be advisable to incorporate an expert-driven e-counseling protocol in order to accommodate participants with greater motivation to change their lifestyle behaviors. |
| Willis | 2017 | original research | Canada | Multi-sectoral Partnerships to Promote Healthy Living and Prevent Chronic Disease (MSP) | Access to stable, trusted and respected networks as a key resource |
| WHO 2015 | 2015 | original research | Spain | Country experiences in integrated policy development for the prevention and control of NCDs | By 2020, increase life expectancy by two years (in good health and free of disability |
| Ministry of Public health | 2014 | strategy document | Korea | National strategic plan for the prevention and control of NCDs in DPR Korea 2014-2020 | Prolong life expectancy in Korea in the period of 2014-2020 by reducing the prevalence of and mortality from NCDs and improving people’s health |
| Baba | 2018 | book | Japan | Integrated stroke prevention program | A substantial decline in the prevalence of severe hypertension and a decline in stroke incidence in the 1960s |
| Iranian National committee for NCDs | 2015 | strategy document | Iran | National document for prevention and control of NCDs and the related risk factors in the Islamic Republic of Iran | Integration of policy making, planning and monitoring of all activities in the area of non-communicable diseases and related risk factors in Iran |
| Pley | 2019 | original research | UK | A health professional's guide to the intersection of public health with intellectual property rights in trade and investment: the case of tobacco plain packaging/ The framework convention on tobacco control | Reduce the brand equity, consumers switching to cheaper products, diminishing industry profits in the long term, delay and complicate the process of purchasing |
| Esdaile | 2019 | original research | UK, Ireland, Canada, New Zealand | National policies to prevent obesity in early childhood | Population wide obesity prevention, childhood obesity prevention |
| Charoenca | 2018 | original research | Thailand | Framework Convention on Tobacco Control/ using an assessment tool | Declining smoking prevalence |
| Wheeler | 2020 | Original research | Canada | Ontario Cancer Plan IV | Improved care, processes, outcomes, and patient experiences |
| Wu | 2017 | original research | Japan | Health and medical service Act, long term care insurance Act, Health promotion Act | Great progress in primary prevention of NCDs |
| Oien | 2013 | review | Norway | NCD strategy 2013- 2017 | Help to ensure that all those who are affected suffer as little as possible and have the progress of their diseases limited, so that they can live a good, long life in spite of their diseases |
| Barber | 2015 | original research | UK | weight management support via the workplace | There was enthusiasm for a weight management service and employees offered membership to SW via the workplace achieved significant weight loss during the 12 week trial period. |
| Riangkam | 2021 | original research | Thailand | mobile health diabetes self-management program | Mobile Health Diabetes self-management program enabled participants to enhance diabetes self-management skill and continuously perform DSM behaviors in their daily life to improve glycemic control. |
| Pattanapongsa | 2019 | original research | Thailand | Use of Facebook as a platform to deliver weight loss intervention | The results of BMI and WHtR reduction in the experimental group were statistically and significantly different in the first four months in comparison with the control group. The result of the study proves that health education and support services through Facebook can be effectively used for weight reduction among students at a public university in Thailand |
| Chalermrueangrong | 2019 | original research | Thailand | Motivation program to quit smoking | This motivational program to quit smoking had a promising outcome regarding smoking abstinence and smoking reduction. |
| Sukpattanasrikul | 2021 | original research | Thailand | Self-management program for older adults with uncontrolled hypertension | the SMP, improved the self-care behavior, decreased blood pressure, and improved the quality of life among older adults with uncontrolled hypertension. |
| Ministry of public health | 2017 | strategic plan | Thailand | 5-year national NCDs prevention and control plan (2017-2021) | Reduce the avoidable burden of illness, death, and disability results from NCDs by means of cooperation between various alliance networks and collaboration on a national, regional, and global level |
| Willis | 2015 | original research | Canada | Interorganizational network to prevent Chronic Diseases in Canada | Enhanced learning, improved use of resources, enhanced or increased relationships, improved collaborative action, network cohesion, improved system outcomes, improved population health outcomes, improved practice and policy planning and improved intersectoral engagement. |
| Buonocore | 2016 | Original research | UK | Regulation and labelling of electronic cigarettes | Apart from few instances, the majority of e-liquids and e-cigarettes manufacturers/distributors do not fully comply with currently enforced regulations, namely CHIP4, CLP, Electrical Equipment (Safety) Regulations of 1994, WEEE and RoHS |
| Choi | 2014 | Original research | Korea | Korean cigarette tax increase | Lower income smokers cut back on smoking more than higher income smokers implies that the additional tax revenue from cigarette tax increases mostly comes from higher income groups as a whole |
| Erinoso | 2020 | original research | Canada | Policies which prohibit tobacco product flavor | Reduce the appeal and use of tobacco products |
| Gore | 2012 | original research | Canada | Healthy eating, physical activity, and healthy weights programs | Explore health disparities and interactions among the social determinants of health to improve the well-being of populations |
| Fukui | 2019 | special report | Japan | Kidney Disease Control Commission Meeting report | Prevent Chronic kidney disease exacerbation mainly by early referrals to nephrologists using “criteria for referral from a primary care physician to a kidney specialist/specialized medical institution” |
| Park | 2019 | Original research | Korea | Role play or standardized patients module of smoking - cessation counseling training for medical student | medical students' smoking cessation knowledge increased. |
| Dubray | 2009 | original research | Canada | Smoke free Ontario act (SFOA) | Within a short time period following implementation of a tobacco point of sale legislation, vendor compliance with such restrictions was very high across vendor trade classes |
| Vanderlee | 2012 | original research | Canada | Canadian Labelling Regulation | The government-mandated nutrition labelling practices are confusing to Canadians. Very few individuals were able to use the information in the Nutrition Facts Table to calculate calorie content when there was more than one serving per container. |
| Bhawra | 2018 | Original research | Canada | the Healthy Eating Strategy | Young Canadians are supportive of nutrition-related policies particularly those pertaining to labelling on menus and food packages, school focused policies, and maximum salt levels for packaged products. |
| Partanen | 2019 | Original research | Finland, Sweden, Denmark | Nord Screen Project | This database is used especially for quality assurance and improvement of cancer screening programs |
| Berridge | 2021 | Policy case study | UK | Electronic nicotine delivery system | Tobacco harm reduction and its usage for smoking cessation. |
| Gatellier | 2020 | Original research | Japan, Korea, Thailand, Singapore | the Asian National Cancer Centers Alliance (ANCCA) | Achievement of the Alliance include the Asia Tobacco-Free Declaration, the establishment of the ANCCA Constitution in 2014 as well as the creation an official website |
| Vergeer | 2019 | Original research | Canada | The Canadian Children's Food and Beverage Advertising Initiative (CAI) | while some food companies are taking strides towards limiting marketing to children on company websites, voluntary industry commitments are not uniformly preventing packaged food, beverage, and restaurant companies in Canada from promoting unhealthy food and beverage products to children. |
| Asukai | 2005 | Commentary | Japan | Japan tobacco policies | Japan's large smoking population, its policies, and its lack of strict regulations surrounding the tobacco industry all attest to the weak policy approach to controlling tobacco |
| Bernstein | 2018 | Original research | Canada | Nutrition Labelling regulations | The study shows the strength of using free sugar DV labelling to discourage the selection of products with excess free sugars |
| Clancy | 2009 | original research | EU countries | price increase and taxation on cigarette/ smokefree workplace/ Regulation of tobacco as a product/ Education and public campaigns/ Smoking cessation | 10% increase in price results in a 4% decrease in consumption/ in Ireland, it was obvious in the first year of smokefree workplaces that there was a dramatic reduction in the overall sales of tobacco. |
| Devi | 2010 | Original research | UK | the standards for school food other than lunch | New standard affected vending machines, 81 percent of vended foods and drinks are no longer permitted under the new standards/ school meals consumption has been decreasing in secondary schools since the introduction of the food-based standards |
| Giesbreght | 2007 | original research | Canada | Focus Community Project | Increase the capacity of health promoters through training, organizing and collective efforts, providing common service |
| Giesbreght | 2007 | original research | Canada | Heart Health Action Program | Integrate the heart health into the existing community health system |
| Givel | 2007 | research article | Canada | National Strategy to Reduce Tobacco Use/ anti-tobacco legislation / the Tobacco Products Control Act | It is an effective approach to reducing tobacco consumption. |
| Ministry for Health | 2010 | national strategy | Malta | A strategy for the prevention and control of NCDs in Malta | Develop a multifactorial approach to NCD prevention through tackling common risk factors targeting both at a population level and also high risk groups |
| WHO Europe regional office | 2016 | Original research | EU countries | Action plan for the prevention and control of NCDs in the WHO European Region | Take integrated action on risk factors and their underlying determinants across sectors and strengthen health systems for improved prevention and control of NCDs |
| Ministry of Public health | 2017 | strategic plan | Thailand | Thailand healthy lifestyle strategic plan phase II, 5 year NCDs prevention and control plan | Relieve the avoidable burden of morbidity; mortality and disability due to non-communicable diseases |
| Iso | 1998 | Original research | Japan | hypertension control program on stroke incidence and prevalence | the study showed a larger decline in stroke incidence and prevalence in Japanese community that received a full range of community wide hypertension interventions. |
| Ministry of health | 2017 | program report | New Zealand | New Zealand Childhood Obesity Program | Reduce the risk of children becoming obese, and increase support for children and young people who are already obese |
| Stubbs | 2013 | original research | UK | primary care/ commercial weight management organization partnership scheme (Slimming World) | The program is as effective for people with high BMIs as for those who are less overweight, and attendances were similar between BMI categories |
| Rujiwatthanakorn | 2011 | original research | Thailand | self-care management program for Thais with essential hypertension | The self-management program was effective in improving knowledge of self-care demands, some self-care ability and blood pressure control in Thais with uncontrolled essential hypertension. |
| Puska | 2002 | original research | Finland | North Karelia Project | Smoking has greatly reduced and dietary habits have markedly changed. The dietary changes have led to reduction in the mean serum cholesterol level of population. Elevated blood pressures have been brought well under control and leisure time physical activity has been increased. |
| Ministry of Health Promotion | 2010 | guidance document | Canada | Healthy eating, physical activity and healthy weights programs | Assist the staff of boards of health to identify issues and approaches for local consideration and implementation of standards |
| Ministry of health | 2010 | strategic plan | Canada | Sodium reduction strategy for Canada | Improving the health of Canadians, addressing individual factors alone by encouraging Canadians to cook with less salt and add less at the table and reduce their sodium levels to an acceptable level |
| Brett | 2020 | Original research | UK | Electronic cigarettes | Not recommending e-cigarettes is associated with a lack of knowledge regarding smoking cessation and e-cigarettes, lack of engagement in smoking cessation practices with patients that smoke, low belief in effectiveness of e-cigarettes, low belief in evidence around e-cigarettes, and not feeling comfortable discussing e-cigarettes with their patients |
| Thiabpho | 2018 | Original research | Thailand | lifestyle modification program. | Systolic and diastolic blood pressures, fasting blood sugar, and waist circumference were significantly improved. |
| Young Park | 2021 | Original research | Korea | smoking cessation consultation program | the inpatient smoking cessation program was associated with a high abstinence rate. Most patients-maintained cessation without medication, suggesting that initial admission, along with a cancer diagnosis, can provide enough motivation to abstain from smoking. |
| leigh | 2012 | article | Canada | Physical Activity Opportunities | The lack of legislation regarding physical activity in childcare centers may greatly contribute to the decrease in physical activity among this young population, only further perpetuating Canada’s childhood obesity epidemic |
| Gilmore | 2010 | Original research | Canada | tobacco price regulation | It could offer a means of preventing downtrading to cheaper products and controlling unwanted industry practices such as cigarette smuggling, price fixing and marketing to the young. |
| Doan | 2020 | Original research | Singapore | e-cigarette | Regardless of the country informing the transition probabilities to and from e-cigarette use in Singapore, a laissez-faire e-cigarette policy could reduce the smoking prevalence in the short term, but it is not as effective as other policies in the long term. |
| Brown | 2021 | Original research | UK | e-cigarette regulation through the EU Tobacco Products Directive | TPD policy roll-out was largely perceived positively and as having been effectively implemented. |
| Okamura | 2014 | Reviewing article | Japan | Worksite Wellness | This approach has successfully contributed to the extremely low incidence of coronary artery disease among Japanese workers |
| Chongthawonsatid | 2017 | Original research | Thailand | The use of a pedometer with or without a supervised exercise program | Using repeated measures analysis found that interaction effect groups and times were significantly different for mean systolic blood pressure, body mass index, hip circumference, and high-density lipoprotein. |
| Norm | 2011 | Reviewing article | Canada | Reducing Dietary Sodium | The Federal Government responded by striking a Intersectoral Sodium Work Group to develop recommendations on how to implement Canada's dietary reference intake values for dietary sodium and by developing timelines and targets for foods to be reduced in sodium, assessing key research gaps with funding for targeted dietary sodium based research, developing plans for public education and for conducting evaluation of the program to reduce dietary sodium. |
| Seo | 2020 | Original research | Korea | The Use of Mobile Personal Health Records | the continuous use of PHRs improved diabetes management outcomes. In addition, the HbA1c reduction pattern was prominent in the PHR continuous user group. |
| Choi | 2021 | Original research | Korea | Community-Based Interventions on Medication Adherence and Hospitalization for Elderly Patients with Type 2 Diabetes at Primary Care Clinics (CRMHDP) | CRMHDP could successful in improving the management of type 2 diabetes mellitus among elderly people in South Korean primary care settings. |
| Potvin Kent | 2014 | Original research | Canada | Children’s food and beverage advertising initiative | Despite improvements in the volume of spots on children’s specialty channels, children’s exposure to food and beverage advertising has increased since the implementation of the CAI. The current self-regulatory system is failing to protect children from food marketing high in fat, sugar and sodium on television. Government regulation needs to be considered. |
| Sandford | 2003 | Reviewing article | UK | Total ban on tobacco advertising and promotion; restrictions on smoking in public places and in the workplace; sustained increases in tobacco taxation combined with measures to curb smuggling; large, bold health warnings on tobacco products; smoking cessation and health education campaigns; and the regulation of tobacco to standards agreed by the health community rather than those set by the tobacco industry. | Given the enormous burden that smoking places on health services, governments in developed nations have generally responded by introducing a range of tobacco control measures. |
| Saitoh Aoki | 2020 | Original research | Japan, Korea, Thailand | National screening programs (NCSP) | Uptake rates of NCSP vary from 5.0%–59.7%. Many women in low- and middle-income countries still do not participate in NCSP. |
| Ow Yong | 2021 | Original research | Singapore | War on Diabetes (WoD) to rally a whole of- nation effort to reduce diabetes burden in the population | The WoD policy generated a sense of unity and purpose across most policy actors. Policy actors were cognizant of the thrusts of the policy and have begun to make shifts to align their interests with the government policy. |
| STOCKWELL | 2020 | Reviewing article | Canada | Cancer warning labels on alcohol containers | Placing cancer warnings on alcohol containers could make a pivotal difference in motivating both drinkers to consume less and regulators to introduce more effective policies to reduce the serious harms of alcohol consumption. |
| SWEANOR | 2003 | Reviewing article | Canada | Legal strategies to reduce tobacco-caused disease | A range of measures impact on the accessibility of tobacco products, the provision of information for consumers, protection of the health and rights of non-users and the significant potential health gains from regulation of tobacco products. |
| den Herder-van der Eerden | 2017 | Original research | German, Ireland, Spain, UK | Accessible integrated palliative care (IPC) | IPC implementation efforts involved a multidisciplinary team approach and cross-sectional coordination. Informal professional relationships, basic medical education and general awareness were regarded as facilitators of IPC. |
| Critchlow | 2020 | Original research | UK | mass media to subtle marketing. Television, social media and special offers are the marketing activities reported most frequently | the restrictions proposed by the UK government are likely to help reduce HFSS consumption |
| Bastos | 2010 | Reviewing article | European Union countries,  Iceland, Norway | Cervical cancer screening programs, national HPV vaccination policies, bans on smoking in public places, colorectal cancer screening programs | There is wide international heterogeneity in cancer control structures in Europe. This provides considerable scope and motivation for cooperation and sharing of experience |
| Son | 2019 | Original research | Korea | Improving medication adherence for elderly patients with hypertension | The intervention program encouraged elderly patients with hypertension to receive continuous care. |
| Choo | 2020 | Original research | Korea | Effects of the healthy children, healthy families, healthy communities’ program for obesity prevention | The intervention group showed significant improvements in total composite scores of healthy-lifestyle behaviors, but not in obesity status among children. Moreover, the intervention group showed significant improvements in parenting behaviors among parents |
| O’Kane | 2020 | Original research | UK, Ireland | Effectiveness of a peer-led school-based walking intervention (WISH) on adolescent girls’ physical activity | The WISH Study will examine the effectiveness of a low-cost, school-based, peer-led walking intervention in increasing physical activity in adolescent girls when delivered across the school year. |
| Rehm | 2017 | Reviewing article | European Union | Increasing in screening for hypertension and drinking, conduct of clinical management of less severe alcohol use disorders for incident people with hypertension in primary healthcare | The implementation of the outlined recommendations could contribute to reducing the burden associated with hypertension and hazardous and harmful alcohol use and thus to achievement of the NCD targets. Implementation should be conducted in controlled settings with evaluation, including, but not limited to, economic evaluation |
| Nygard | 2007 | Original research | Norway | The effect of the antepartum Pap smear on the coverage of a cytological CC screening program. | 69% of the pregnant women had a Pap smear during one year of follow-up since beginning of the pregnancy with the majority taken during the antepartum period. |
| Wanderley | 2010 | Original research | Portugal | Effects of a moderate-intensity walking program on blood pressure, body composition and functional fitness in older women | Data demonstrated a mean reduction of 12mmHg in the women’s systolic blood pressure; improvements of 5 repetitions on the muscular endurance of the lower limbs; however, there were no significant alterations after the walking program on body composition variables |
